# Emphysema Is Common in Lungs of Cystic Fibrosis Lung Transplantation Patients: A Histopathological and Computed Tomography Study

**DOI:** 10.1371/journal.pone.0128062

**Published:** 2015-06-05

**Authors:** Onno M. Mets, Suzan M. Roothaan, Inez Bronsveld, Bart Luijk, Ed A. van de Graaf, Aryan Vink, Pim A. de Jong

**Affiliations:** 1 Department of Radiology, University Medical Center Utrecht, Utrecht, The Netherlands; 2 Department of Pathology, University Medical Center Utrecht, Utrecht, The Netherlands; 3 Department of Respiratory Medicine, University Medical Center Utrecht, Utrecht, The Netherlands; Central Michigan University School of Medicine, UNITED STATES

## Abstract

**Background:**

Lung disease in cystic fibrosis (CF) involves excessive inflammation, repetitive infections and development of bronchiectasis. Recently, literature on emphysema in CF has emerged, which might become an increasingly important disease component due to the increased life expectancy. The purpose of this study was to assess the presence and extent of emphysema in endstage CF lungs.

**Methods:**

In explanted lungs of 20 CF patients emphysema was semi-quantitatively assessed on histology specimens. Also, emphysema was automatically quantified on pre-transplantation computed tomography (CT) using the percentage of voxels below -950 Houndfield Units and was visually scored on CT. The relation between emphysema extent, pre-transplantation lung function and age was determined.

**Results:**

All CF patients showed emphysema on histological examination: 3/20 (15%) showed mild, 15/20 (75%) moderate and 2/20 (10%) severe emphysema, defined as 0–20% emphysema, 20–50% emphysema and >50% emphysema in residual lung tissue, respectively. Visually upper lobe bullous emphysema was identified in 13/20 and more diffuse non-bullous emphysema in 18/20. Histology showed a significant correlation to quantified CT emphysema (p = 0.03) and visual emphysema score (p = 0.001). CT and visual emphysema extent were positively correlated with age (p = 0.045 and p = 0.04, respectively).

**Conclusions:**

In conclusion, this study both pathologically and radiologically confirms that emphysema is common in end-stage CF lungs, and is age related. Emphysema might become an increasingly important disease component in the aging CF population.

## Introduction

Cystic fibrosis (CF) is a recessive genetic disease, caused by a mutation in the cystic fibrosis transmembrane conductance regulator (CFTR) gene, affecting many organ systems throughout the body. The predominant negative prognostic factor in CF is chronic progressive lung disease [1]. Lung disease in cystic fibrosis involves decreased mucociliary clearance, repetitive pulmonary infections, inflammation and ultimately bronchiectasis leading to reduced lung function, loss of quality of life and premature death [2].

Until recently, the role of emphysematous changes in CF lung disease has been considered to be minimal. In the 1970’s and 1980’s several postmortem studies reported some visually detected emphysema in older CF patients in addition to bronchiectasis, mucoid impaction, and fibrosis [3–8]. Recently it has been shown that next to airway surface dehydration with chronic mucus obstruction and inflammation, also emphysema occurs in mice with CF lung disease [9, 10].

Over time disease management in CF has changed with new therapeutic options, resulting in a dramatically improved survival. Given this increased survival in those who have benefited, emphysema may have become a more prominent disease component in CF. This was recently also reported by Wielputz et al. who demonstrated that non-invasive CT measurements of emphysema increase with age and correlate with lung function parameters [11], suggesting that emphysema contributes to disease severity in CF. However, to our knowledge no studies have histologically assessed the presence and extent of emphysema in today’s CF patients, nor related this to quantitative CT findings. Therefore, this study aims to investigate the presence and extent of emphysema in explanted CF lungs using both histology and CT densitometry.

## Methods

### Ethics statement

Need for informed consent was waived by the institutional ethics committee of the University Medical Center Utrecht. Specific approval of the ethics committee was not necessary for this study, since all histology and radiology was part of the routine diagnostic procedure. Material was handled in a coded way that met the criteria of the code of conduct for responsible use of human tissue that is used in The Netherlands for the use of human tissue in medical research (www.federa.org).

### Study population

Out of all patients that underwent lung transplantation between 2004 and 2009 in the University Medical Center Utrecht (n = 144) we selected those suffering from CF and of whom histological material of the explanted lungs and pre-operative computed tomography (CT) was available. This resulted in 24 subjects. Four subjects were excluded due to failure of the automated CT quantification method, resulting in a study population of 20 subjects. Clinical and lung function data were collected and structural lung disease was visually scored in this pre-transplant population. Scoring was performed according to a modified structured CT scoring method [12] by a thoracic radiologist with >10 years of experience, who previously showed good agreement with other observers [13]. The scoring system assesses several disease components on airways, mucus plugging and parenchyma on a 3-points scale on a per lobe basis. A total lung score was calculated using data for all six different lobes (lingula is considered a separate lobe) using a standardized summation equation [12]. Scores are presented as actual value and percentage of total possible score. For the present study we slightly adjusted the original CT scoring method by excluding air trapping and the parenchymal cyst/bullae parameter, but adding emphysema scoring based on the exact same principle; bullous and non-bullous emphysema was scored per lobe using a 3-point scale; 0 = none; 1 = <1/3; 2 = 1/3 to 2/3; 3 = >2/3 of the lobe affected.

### Lung function testing

Postbronchodilator spirometry was performed with ZAN equipment (ZAN messgerate GmbH), according to European Respiratory Society guidelines [14]. Forced expiratory volume in the first second (FEV_1_) and the ratio of FEV_1_ to forced vital capacity (FEV_1_/FVC) are expressed as percent predicted [15], and the median flow between 25%–75% of FVC (MEF_25–75_) is expressed as percentage.

### Histopathology

**Fig 1:**
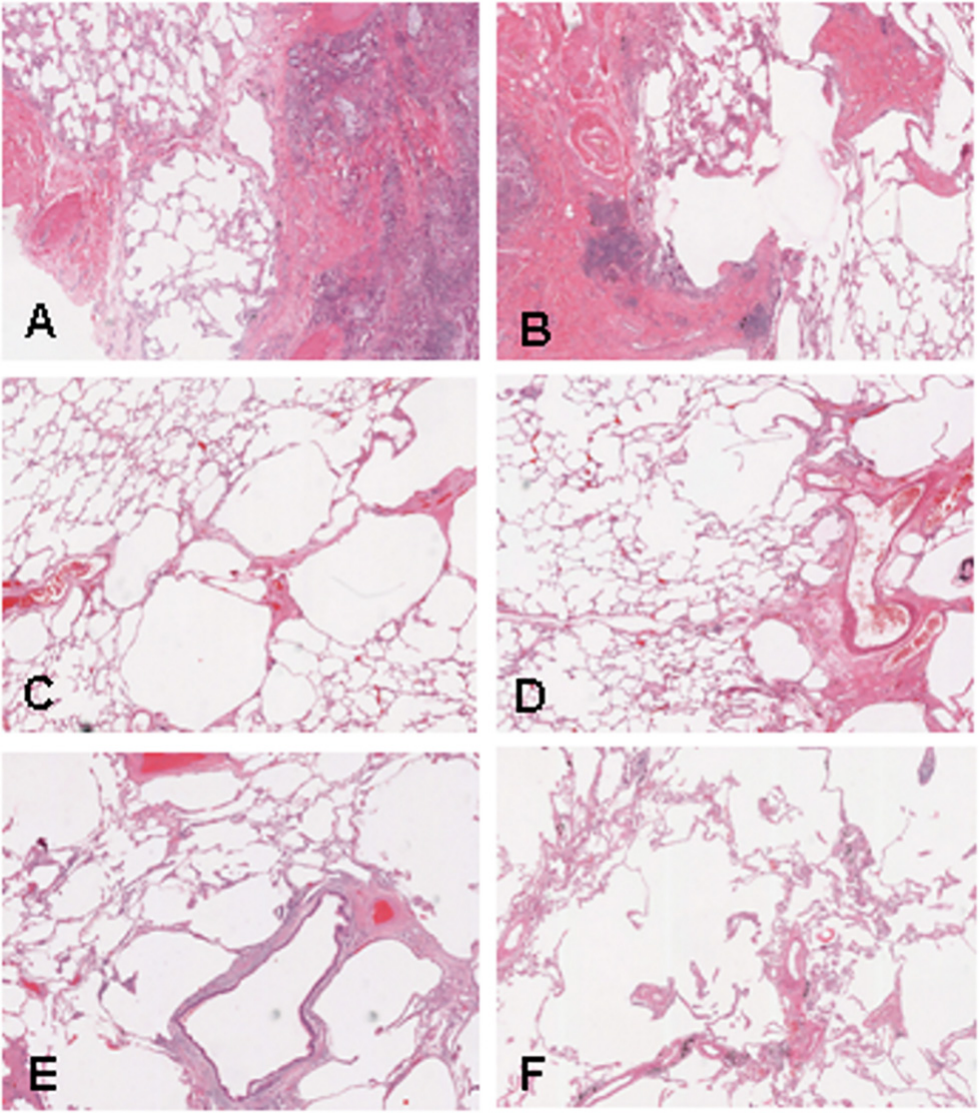
Examples of emphysema in the explanted lungs (H&E stained slides, magnification 200x). A-E: Emphysematous changes in patients with CF. A = score 1: some emphysematous changes are present next to a scar in <20% of residual lung tissue. B-D = score 2: emphysematous changes in 20–50% of residual lung tissue, next to scar tissue (B) or in the paraseptal region (C and D). E = Score 3: emphysematous changes in >50% of residual lung tissue. F = Emphysema in an explanted lung of a patient with severe COPD, for comparison.

After formalin inflation fixation, the explanted lungs were processed according to a standardized protocol. From each lobe a peripheral and central sample of 2 x 1 cm was taken for diagnostic purposes. For the histological assessment of emphysema, all the Hematoxylin and Eosin (HE) stained slides from the pathology archive were reviewed. Emphysema was defined as lung tissue characterized by abnormal, permanent enlargement of the air spaces distal to the terminal bronchiole, accompanied by destruction of their walls and without obvious gross fibrosis [16]. Alveolar destruction was recognized by the finding of “free-floating” pieces of viable alveolar septa [17]. The amount of emphysema in the residual lung tissue was scored in consensus by two observers, using a semi-quantitative scoring method. A 4-point scale was used: score 0 (no emphysematous changes), score 1 (0–20% emphysema), score 2 (20–50% emphysema) and score 3 (>50% emphysema), which is illustrated in Fig 1. Areas of established fibrosis or bronchiectasis were excluded from the estimation. The observers were blinded from the CT emphysema measures.

### Quantitative Computed Tomography

In the workup to lung transplantation inspiratory CT of the thorax was obtained. Scans were performed at multi-detector CT scanners of the same vendor in use in our hospital (Brilliance 16P (N = 4), Brilliance 40 (N = 1), Brilliance 64 (N = 8) and Mx8000IDT (N = 7); Philips Medical Systems, Cleveland OH). Scan parameters were 120 kVp at 100–150 mAs. Thin slices were reconstructed (0.9 or 1.0 mm) at 0.45 to 0.7 mm increment using a sharp reconstruction algorithm (Philips C-filter (15/20 subjects) or YC-filter (5/20 subjects)).

**Fig 2:**
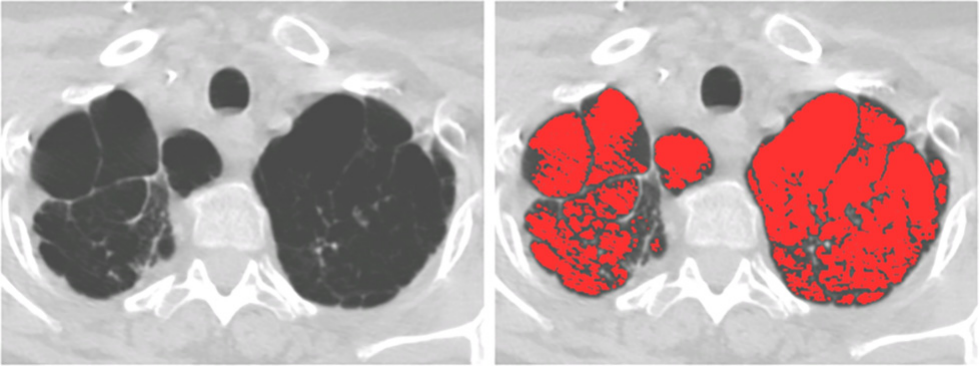
Pre-transplant axial CT image of CF patient in the work-up to lung transplantation. Both lungs show substantial apical bullous emphysema (panel A), automatically quantified (panel B) as 17,9% (IN-950). Histopathologically this case was assigned score 2 (20–50% emphysema in the residual lung tissue).

CT emphysema was quantified using custom software (Emphylx; Department of Radiology/iCAPTURE Laboratory, University of British Columbia; Vancouver, BC, Canada). Briefly, each lung was segmented from the large vessels and chest wall using CT values of -1,000 to -500 Hounsfield Units (HU). The total lung volume was calculated by summing the voxel dimensions in each slice. CT emphysema was quantified using a commonly used measure for emphysema quantification; percentage of voxels below -950HU in inspiration (IN_-950_) [18–20], see Fig 2.

### Statistics

Correlation between quantitative CT emphysema, visual CT emphysema and the histopathology score, as well as between CT emphysema and age was assessed using the non-parametric Spearman’s Rho test.Analysis was performed using SPSS statistical software package version 20.0 (SPSS,Chicago, Illinois.). A p-value <0.05 was considered significant.

## Results

### Study population

The study population comprised 20 CF patients that underwent bilateral lung transplantation (12 male, 8 female). Median age was 32.0 years (IQR 27.5–42.6). Median time interval between the CT and explantation of the lungs was 11.1 months (IQR 8.1–13.3). Median time interval between the CT and lung function testing was 2 days (IQR 1–4).

Table 1 summarizes the clinical data of the study population. Most subjects showed a homozygote F508del mutation and 19/20 had Pseudomonas colonization. As can be expected in an end-stage lung disease population, pre-transplantation lung function parameters were very low. FEV_1_ showed a median of 23.5% (IQR 20.3–25.8) of predicted. For FEV_1_/FVC this was 44.5% (IQR 40.3–57.8) of predicted, while the median MEF_25–75_ was 7% (IQR 6–7).

**Table 1:** Clinical data of the study population.

Case	Sex	Age	Genotype	Pancreatic status	CFRD	Pseudomonas colonization	CT quantified Lung volume (L)	CT Emphysema (IN_-950_, %)	FEV_1_ (%pred)	FEV_1_/FVC (%pred)	MEF_25–75_ (%)
1	F	30,8	F508del/F508del	Exocrine insufficiency	Yes	Yes	4,80	10,3	21%	46%	5%
2	F	34,3	F508del/F508del	Exocrine insufficiency	Yes	Yes	4,88	15,8	22%	43%	7%
3	M	37,0	*	Normal	No	Yes	5,49	10,6	35%	43%	6%
4	M	44,0	F508del/3272-26 A>G	Normal	No	Yes	4,66	10,4	24%	58%	9%
5	F	20,5	*	Exocrine insufficiency	Yes	Yes	4,32	2,7	22%	65%	7%
6	M	44,0	*	Exocrine insufficiency	No	Yes	5,81	11,3	18%	47%	6%
7	M	31,5	F508del/F508del	Exocrine insufficiency	Yes	Yes	7,16	8,6	18%	24%	5%
8	F	27,4	F508del/F508del	Exocrine insufficiency	Yes	Yes	3,57	8,9	23%	68%	10%
9	M	32,2	F508del/F508del	Exocrine insufficiency	Yes	Yes	7,37	7,6	22%	45%	6%
10	M	38,4	F508del/A445E	Exocrine insufficiency	No	Yes	5,95	5,2	25%	51%	6%
11	M	35,6	F508del/R553X	Exocrine insufficiency	Yes	Yes	6,97	4,8	26%	41%	7%
12	F	31,7	F508del/F508del	Exocrine insufficiency	Yes	Yes	5,06	10,8	24%	54%	8%
13	F	27,8	F508del/F508del	Exocrine insufficiency	Yes	Yes	6,56	17,7	21%	35%	5%
14	M	53,8	R347P/4382delA	Exocrine insufficiency	No	Yes	8,68	16,5	21%	27%	5%
15	M	21,8	F508del/F508del	Exocrine insufficiency	No	Yes	6,92	5,1	15%	49%	5%
16	F	48,6	F508del//R1162X	Normal	No	Yes	4,89	17,9	24%	51%	6%
17	M	22,5	F508del/F508del	Exocrine insufficiency	Yes	Yes	5,99	5,7	20%	61%	5%
18	V	16,2	F508del/R1162X	Exocrine insufficiency	No	Yes	4,75	1,2	29%	63%	12%
19	V	53,1	F508del/3272-26 A>G	Normal	No	No	8,11	7,5	22%	35%	6%
20	M	28,8	*	Exocrine insufficiency	No	Yes	4,99	16	20%	58%	8%
All	-	32.0 (27.5–42.6)	-	80% ^a^	50% ^b^	95% ^c^	5.65 (4.85–6.96)	9.5 (5.3–14.7)	22% (20%-24%)	48% (42%-58%)	6% (5%-8%)

Total values are presented as median with interquartile range.

* Missing data (4 cases)

^a^ percentage with exocrine insufficiency

^b^ percentage with CFRD

^c^ percentage with pseudomonas colonization

*IN*_*-950*_ CT emphysema quantified as the percentage of voxels below -950HU in inspiration

*FEV*_*1*_ Forced expiratory volume in the first second; *FEV*_*1*_ */FVC* ratio of FEV_1_ over forced vital capacity; *MEF*_*25–75*_ Mean flow between 25% and 75% of forced vital capacity; *CFRD* cystic fibrosis related diabetes.

Visual grading of structural lung disease is presented in Table 2. Regarding emphysema, 13/20 subjects visually showed upper lobe predominant bullous emphysema. Furthermore, 18/20 showed non-bullous emphysema to some extent. This was mainly diffuse or somewhat lower lobe predominant. In total 10/20 subjects had a total emphysema score of at least six, which means that they had either some emphysema (<1/3 of the lobe) in every lobe, or more extensive emphysema in selected lobes. As anticipated in pre-transplant subjects our population showed severe bronchiectasis, mucus plugging and peribronchial thickening was present in all subjects. The extent of lung disease is represented by the median percentage of total possible scores per disease component and the total modified CT score (bronchiectasis 50%, mucus plugging 42%, peribronchial thickening 46%, and total score 38%); see Table 2.

**Table 2:** Visual grading of structural CF lung disease.

Case	BE ^a^	%BE	MP ^b^	%MP	PBT ^a^ ^,^ ^b^	%PBT	PAR ^a^	%PAR	EMPH ^a^	%EMPH	Total	%Total
**1**	36	50	12	33	30	56	2	6	9	25	79	34
**2**	49	68	12	33	15	28	7	19	0	0	73	31
**3**	36	50	24	67	20	37	3	8	4	11	67	29
**4**	36	50	24	67	38	69	7	19	8	22	93	40
**5**	65	90	18	50	29	54	3	8	0	0	101	43
**6**	31	43	12	33	26	48	4	11	5	14	68	29
**7**	32	44	20	56	30	56	6	17	6	17	77	33
**8**	52	72	24	67	30	56	9	25	2	6	97	42
**9**	29	40	12	33	19	34	6	17	9	25	64	27
**10**	48	67	18	50	23	42	3	8	3	8	80	34
**11**	36	50	6	17	23	42	3	8	2	6	65	28
**12**	34	47	10	28	20	36	10	28	5	14	71	30
**13**	33	46	24	67	23	42	2	6	9	25	71	30
**14**	30	42	18	50	26	48	5	14	13	36	77	33
**15**	23	31	12	33	18	33	4	11	2	6	49	21
**16**	45	63	18	50	28	51	5	14	10	28	91	39
**17**	32	44	12	33	24	44	2	6	7	19	67	28
**18**	53	73	14	39	40	74	2	6	1	3	98	42
**19**	24	33	16	44	23	42	0	0	10	28	59	25
**20**	42	58	12	33	30	56	8	22	12	33	94	40
All	36 (31–47)	50 (43–66)	15 (12–20)	42 (33–54)	25 (21–30)	46 (38–56)	4 (2–7)	11 (6–19)	6 (2–9)	15 (6–25)	75 (67–92)	32 (29–39)

Total scores are presented as median with interquartile range. Scoring is based on Ref 12.

Values are an overall score for all six lung lobes (lingula considered a separate lobe). % scores of each parameter are the percentage of the total score possible.

*BE* bronchieactasis; *MP* Mucusplugging; *PBT* Peribronchial thickening; *PAR* Parenchymal (ie. opacity and groundglass); *EMPH* Emphysema (ie. bullous and non-bullous)

^a^ Components scored on a per lobe basis: None, <1/3, 1/3 to 2/3 or >2/3 of the lung lobe (0–3)

^b^ Components scored on a per lobe basis: None, mild, moderate or severe (0–3)

### Histopathology

All explanted lungs showed emphysematous changes to some degree, ranging from mild focal emphysema to severe destructive emphysema in the majority of the residual lung tissue (Fig 1). The lungs of 3/20 (15%) revealed mild emphysema, 15/20 (75%) moderate emphysema and 2/20 (10%) severe emphysema (Table 3). Most emphysema was distributed in an inhomogeneous pattern, sometimes located around scar tissue.

**Table 3:** Patient distribution in the histopathologically defined subgroups.

	Emphysema score			
	0	1	2	3
Patients, n (%)	0 (0)	3 (15)	15 (75)	2 (10)

Emphysema extent was semi-quantitatively scored by two observers in consensus, using a 4-point scale: score 0 (no emphysematous changes); score 1 (0–20% emphysema); score 2 (20–50% emphysema); and score 3 (>50% emphysema). Areas of established fibrosis or bronchiectasis were excluded from the estimation.

### Quantitative Computed Tomography

**Fig 3:**
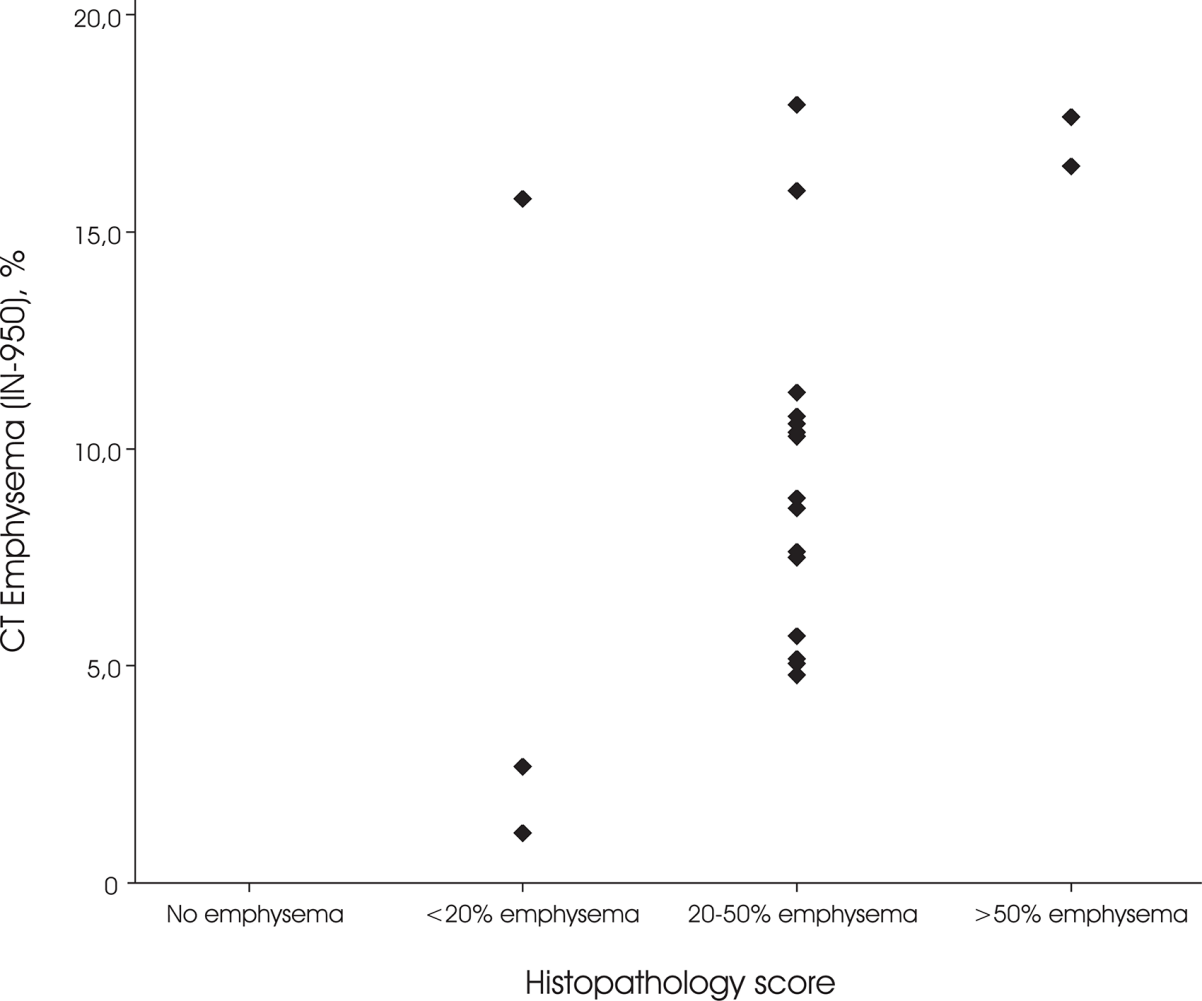
CT emphysema distribution between the semi-quantitative histopathologic emphysema score subgroups. CT emphysema is defined as the percentage of lung volume with an attenuation of -950 Houndfield Unit or lower (IN_-950_).

Median lung volume was 5.65 liter (IQR 4.82–6.96). The median CT emphysema was (IN_-950_) 9.6% (IQR 5.3–14.7). Both automatically quantified CT emphysema (IN_-950_) and visual emphysema scores showed a positive relation to the histopathological scores (R = 0.49, p = 0.03 and (R = 0.69, p = 0.001, respectively). Correlation between quantified CT emphysema and visual emphysema scores was substantial (R = 0.57, p<0.01). There was a single case who showed a mosaic lung pattern which resulted in a high CT emphysema value, but was histopathologically assigned score 1 and visually had no emphysema. Our findings indicate that the subjects with more emphysema were generally indeed also assigned a higher histopathology score (Fig 3).

**Fig 4:**
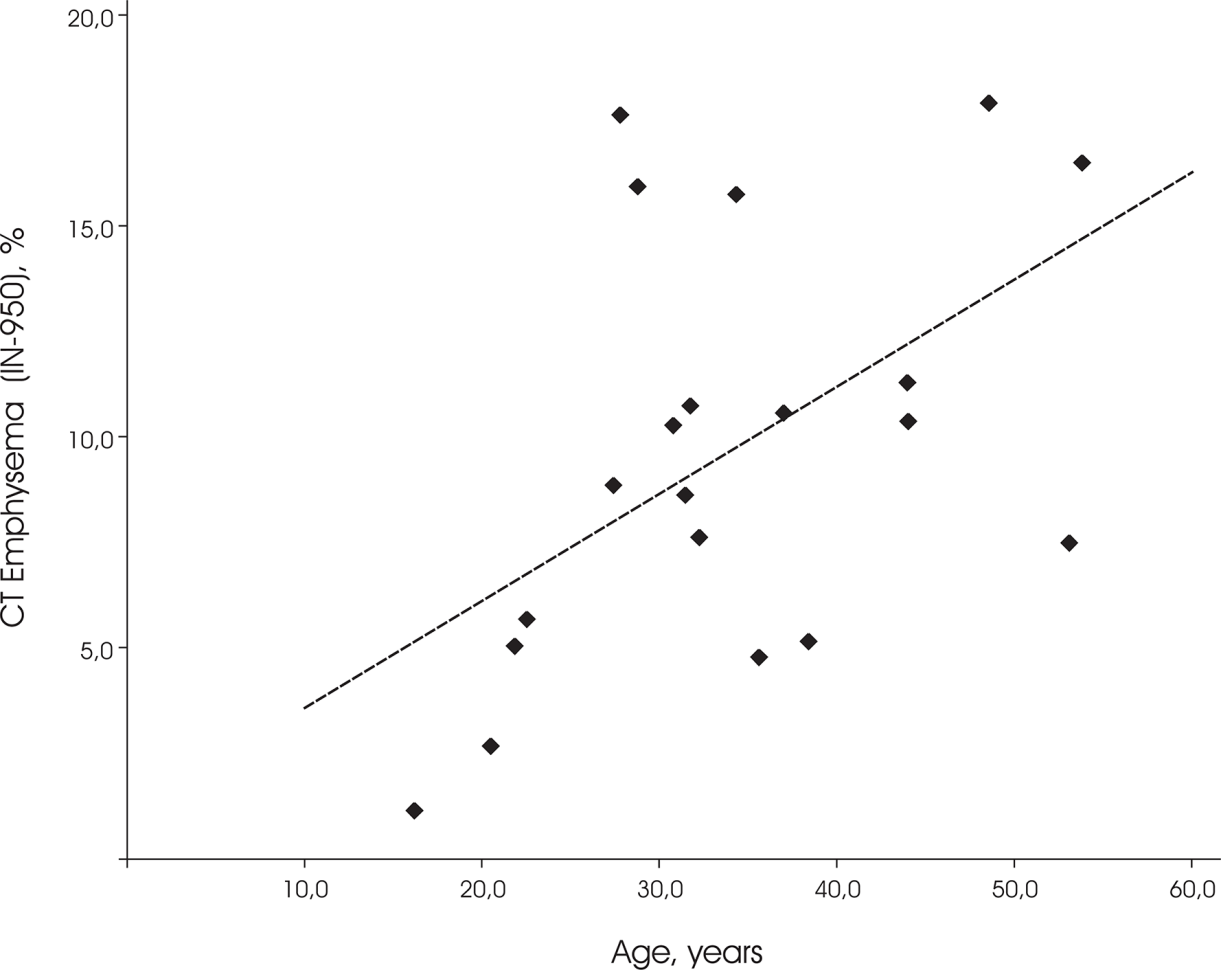
Relation between CT Emphysema extent and age. CT emphysema is defined as the percentage of lung volume with an attenuation of -950 Houndfield Unit or lower (IN_-950_).

Also, age showed a significant positive correlation to automatically quantified CT emphysema (R = 0.45, p = 0.045; see Fig 4) as well as to visual emphysema score (R = 0.47, p = 0.04). Histopathology score and age did not reach significance (R = 0.38, p = 0.10), due to one outlier of 28 years with histopathology score 3.

Lung function parameters in our study population showed significant correlation to histopathology for both FEV_1_/FVC (R = -0.46, p = 0.04) and MEF_25–75_ (R = -0.54, p = 0.02), but not FEV_1_. Correlation between lung function parameters and CT emphysema did not reach significance; see Table 4.

**Table 4:** Correlation between lung function parameters and histopathology and quantified CT emphysema score.

	Histopathology score	CT emphysema (IN_-950_)
FEV_1_ (%pred)	R = -0.24, p = 0.31	R = -0.32, p = 0.17
FEV_1_/FVC (%pred)	R = -0.46, p = 0.04	R = -0.42, p = 0.07
MEF_25–75_ (%)	R = -0.54, p = 0.02	R = -0.42, p = 0.07

*IN*_*-950*_ CT emphysema quantified as the percentage of voxels below -950HU in inspiration; *FEV*_*1*_ Forced expiratory volume in the first second; *FEV*_*1*_ */FVC* ratio of FEV_1_ over forced vital capacity; *MEF*_*25–75*_ Median flow between 25% and 75% of forced vital capacity.

## Discussion

Using pre-transplantation CT-densitometry and histological examination of the explanted lungs, this study demonstrates that emphysema is common in adult cystic fibrosis patients with end-stage disease. These emphysematous changes are additive to the already known changes such as bronchiectasis in CF lungs [1, 21].

From previous studies published in the 1970’s and 1980’s it is known that histologically some, but only minor emphysema can be seen in the lungs of CF patients [3–6]. Bedrossian et al. [3] showed progressively more emphysema among the older patients in a population of 82 deceased CF patients (20 male, 62 female, age ranging from 5 days to 24 years old), but this never involved more than 10% of the lung parenchyma by grid scoring. Further, Sobonya et al. reported mild destructive emphysema in autopsy lungs of 9 patients and argued this was seen only in adults [4]. Regarding emphysema occurrence in CF lungs our results are in line with this previous literature. However, emphysema extent in our study wows more substantial, both quantitatively and histologically. In this study we observed CT emphysema values that are more than 3 times the upper limit of normal in healthy males (with comparable age range) at our scanners [22]. These are thus clearly abnormal. Also, histolocigally destructive emphysema in some cases approached the changes as seen in explanted lungs of COPD patients. This difference to previous literature we believe is likely explained by the fact that life expectancy of CF patients has increased dramatically in the last decades, with a median predicted age of survival in the early 40s nowadays [23]. This explanation is supported by the positive correlation between CT emphysema extent and age.

In a recent study among modern CF patients, Wielputz et al. demonstrated that quantitative CT measurements of emphysema increase with age [11]. Furthermore, they showed that these values correlate with lung function parameters, suggesting that emphysema contributes to disease severity in CF. Our present study confirms their findings on the presence of CT emphysema and its positive relation to age, but moreover, we extent their findings by pathologically confirm emphysema as a disease component in CF. Although we found a significant correlation between the pre-transplantation MEF_25–75_ and FEV_1_/FVC and the histopathology subgroups, we were unable to reproduce the correlation between other lung function parameters and CT emphysema. We believe this may well be due to our smaller study population. Also, our population showed end-stage lung disease with worse and less variable lung function than Wielputz’ population (e.g. FEV_1_ 24% ± 5.1 of predicted versus 46 ± 30 of predicted). Correlation to the destruction of alveolar walls on histopathology for MEF_25–75_ and FEV_1_/FVC but not FEV_1_ might be explained by the fact that in more advanced lung disease they are flow obstruction parameters that represent more distal airways function than FEV_1_. We feel CT emphysema is probably a more global parameter and less accurate, and therefore not correlated. Nevertheless, it should be specifically noted that our results are based on a fairly small study population.

The presence of true destruction of alveolar walls in CF lungs implicates a remodeling process with an irreversible component. A complex network of the innate immune system and inflammatory changes are thought to underlie emphysema development in CF [24]. Recent literature points towards surface dehydration with inflammatory recruitment of immune cells, and an important role for neutrophil elastase and metalloproteinase-12 in emphysema formation [9, 10, 25, 26]. Briefly, due to the CFTR mutation there is malfunction of the transmembrane protein in airway epithelia in CF, leading to ion transport dysfunction. Normally, careful regulation of Na+ and Cl- secretion maintains airway surface fluid and normal mucociliary clearance. In case of CF, the malfunction in ion transportation leads to surface dehydration and dysfunction of the mucociliary apparatus [27]. This leads to increased mucus concentration and plugging, with inflammation and infection, which are the processes that cause the disease-related recurrent infections, bronchiectasis and likely also emphysema development in CF. Possibly, contraction of scar tissue may also play a role in the development of alveolar damage, given that alveolar wall destruction was mostly observed around areas with scars of healed bronchiectasis. However, future research has to unravel the exact pathophysiological mechanism of emphysema development in CF patients.

The major strength of the current study is that we pathologically confirm emphysema as a common disease component in current CF patients, which was previously only suggested based on quantitative CT measurements. Our study also suffers from potential limitations. First, there may be sampling error in histology. According to a standardized protocol two samples for each anatomical lung lobe were scored, which could potentially result in under- or overestimation of emphysema depending on the emphysema distribution. Nevertheless, we showed a positive relation between the histologic and radiologic data, suggesting that the observed microscopic changes provide a good representation of the abnormalities in the entire lung. Second, we semi-quantitatively determined the emphysema in histological slides. A quantitative method might provide more detailed information. However, the validated histological tests such as mean linear intercepts and destructive index are developed for emphysematous lungs without scars and bronchiectasis that probably influence these measurements. Third, several possible confounders of the quantitative emphysema measurements have to be considered, including the relatively sharp kernels that were used, different CT scanners from the same vendor and sacculations and bronchiectasis that were included in the low density voxel analysis. Nevertheless, we found a significant correlation to histology and a trend for visual emphysema score, and judged the influence of these confounders to be limited. Fourth, the study sample size is limited. We feel that this is inherent to the fact that lung transplantation is a very specialized procedure requiring a high-care center, and is only performed in end-stage disease. Nonetheless, we were able to find a significant positive correlation with increasing age. Last, due to the retrospective nature of the study we had to use CT scans clinically obtained in the workup towards lung transplantation. Lung function and CT data were obtained only days apart, with no relevant time interval. Contrarily, it did result in a certain time interval towards the histopathological data. However, since emphysema is irreversible and most likely slowly progressive, this may have only resulted in some underestimation of results.

In conclusion, this study histopathologically and radiologically confirms that emphysema is common in end-stage CF lung disease, and is positively related to age.

## Supporting Information

S1 Dataset Dataset of the visual scoring of disease severity. (XLSX)

S2 Dataset Complete dataset of the study data. (XLSX)
